# Supplementary material for: Chimeric inheritance and crown-group acquisitions of carbon fixation genes within Chlorobiales: Origins of autotrophy in Chlorobiales and implication for geological biomarkers
Source: PLoS One. 2022 Oct 13;17(10):e0275539. doi: 10.1371/journal.pone.0275539 (PMC9560492; doi:10.1371/journal.pone.0275539)
Supplement: S1 Table — (PDF) [file pone.0275539.s001.pdf]

S1 Table. Enzymes, NCBI Accessions, Genome IDs, alignment, taxonomic diversity, and ML tree model for query sequences.

Enzyme Name	Accession	Genome ID	Alignment Length	# Taxa	BIC
malate dehydrogenase	WP_012498653	CP001100.1	318	242	LG+R6
fumarate hydratase	WP_012500396	CP001100.1	480	250	LG+R8
fumarate hydratase	WP_010932514	AE006470.1	565	242	LG+R7
fumarate reductase	WP_012498718	CP001100.1	448	248	LG+R8
succinyl-CoA synthetase	WP_012500801	CP001100.1	426	248	LG+R7
2-oxoglutarate:ferredoxin oxidoreductase, alpha subunit	WP_012500035	CP001100.1	657	243	LG+R7
isocitrate dehydrogenase	ACF14612	CP001100.1	356	232	LG+R8
isocitrate dehydrogenase	WP_010932043	AE006470.1	765	250	LG+F+R7
aconitate hydratase	WP_012500984	CP001100.1	775	249	LG+F+R8
aconitate hydratase	WP_010932230	AE006470.1	889	250	LG+F+R7
ATP citrate lyase alpha subunit	WP_012501083	CP001100.1	658	247	LG+R7
ATP citrate lyase beta subunit	WP_012501083	CP001100.1	443	287	LG+R7
pyruvate:ferredoxin oxidoreductase	WP_012499296	CP001100.1	1357	247	LG+R6
