# Supplementary figures and images for: Chimeric inheritance and crown-group acquisitions of carbon fixation genes within Chlorobiales: Origins of autotrophy in Chlorobiales and implication for geological biomarkers
Source: PLoS One. 2022 Oct 13;17(10):e0275539. doi: 10.1371/journal.pone.0275539 (PMC9560492; doi:10.1371/journal.pone.0275539)

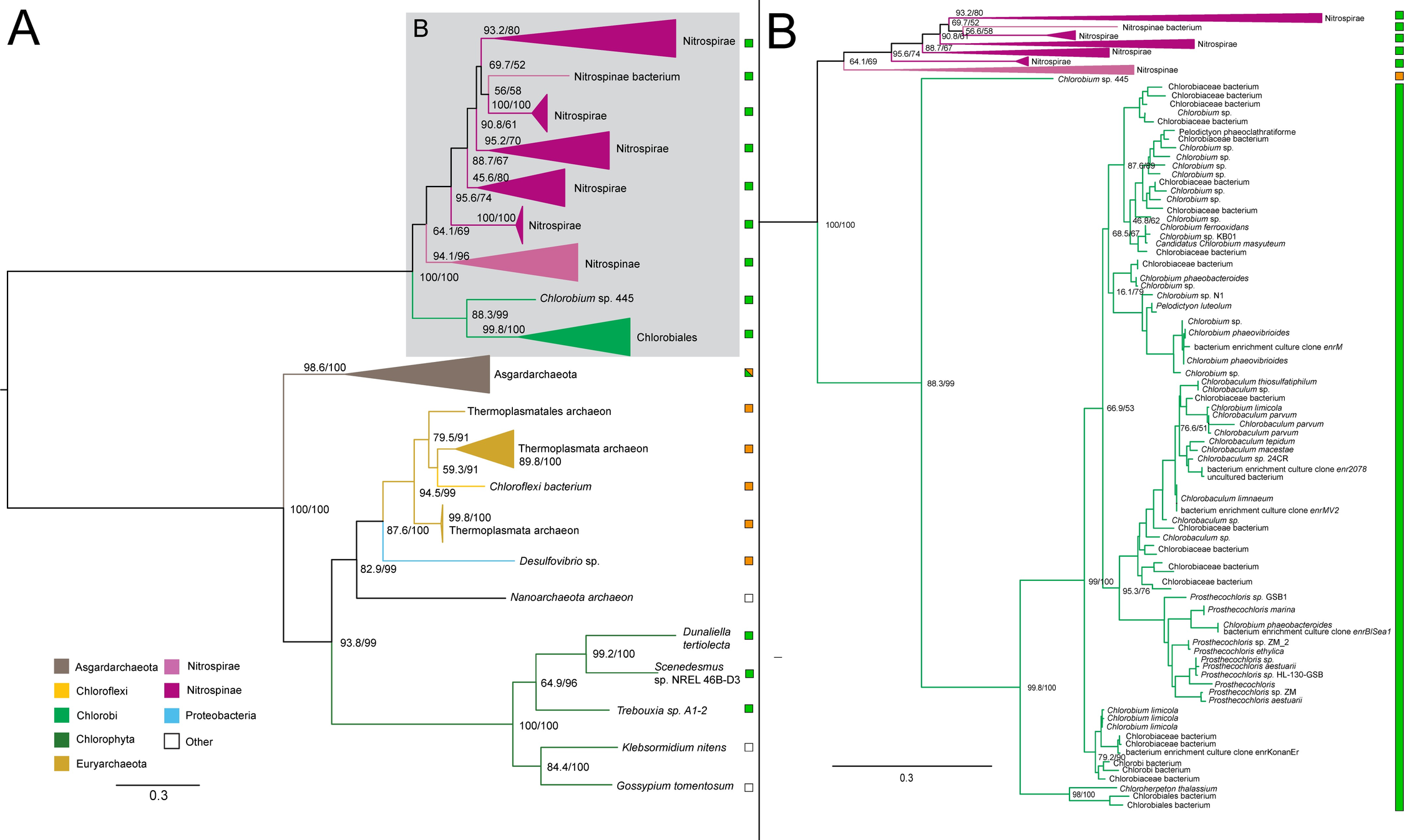

Supplement: S1 Fig — (A) midpoint-rooted tree with collapsed clades labeled with taxonomic group names. (B) Higher resolution tree showing crown Chlorobiales and closely related sequences in Nitrospira/Nitrospinae. Support values indicate approximate likelihood ratio test (aLRT)/ bootstrap (100 replicates). Major clades with bootstrap (BS) support are labeled with respective values. Color bars to the right of the tree indicate autotrophic (green), heterotrophic (orange), or undetermined (white) carbon metabolisms. (TIF) [file pone.0275539.s002.tif]

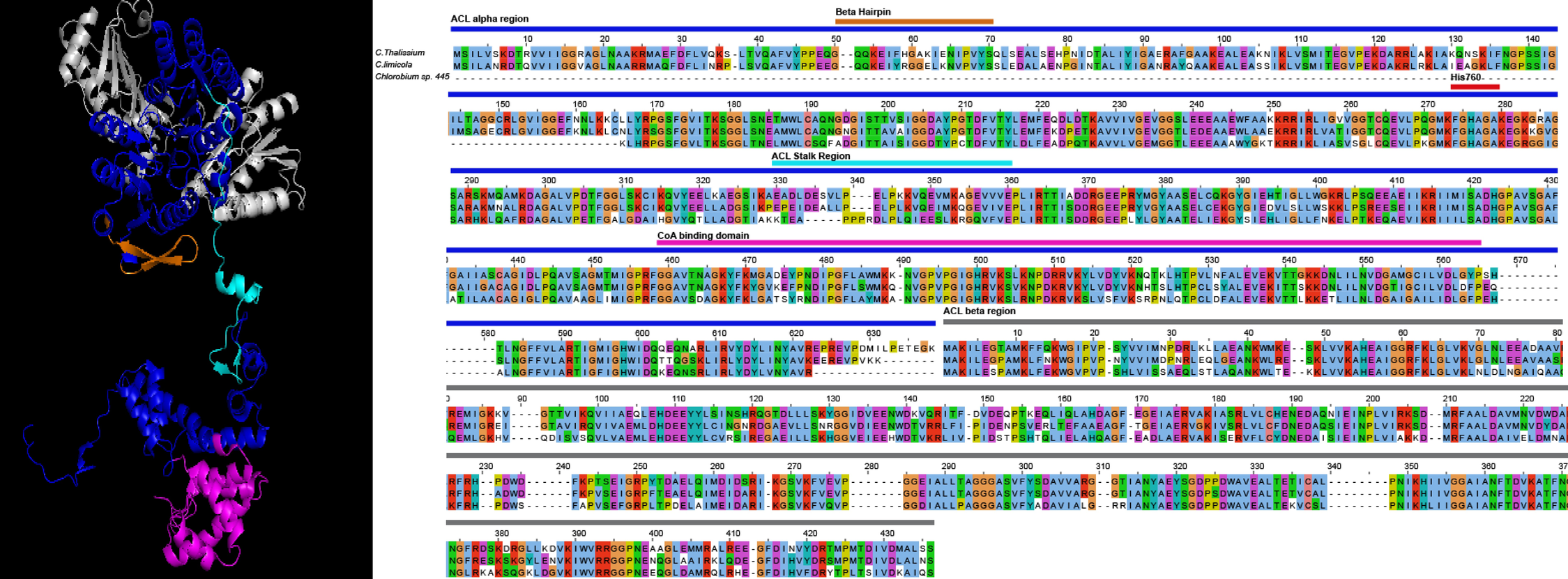

Supplement: S2 Fig — Structural and labeled functional regions regions are colored corresponding to bars above sequence alignments. The amino acid alignment includes are sequences from C.thalassium, C.limicola, Chlorobium sp.445. [23]. (TIF) [file pone.0275539.s003.tif]

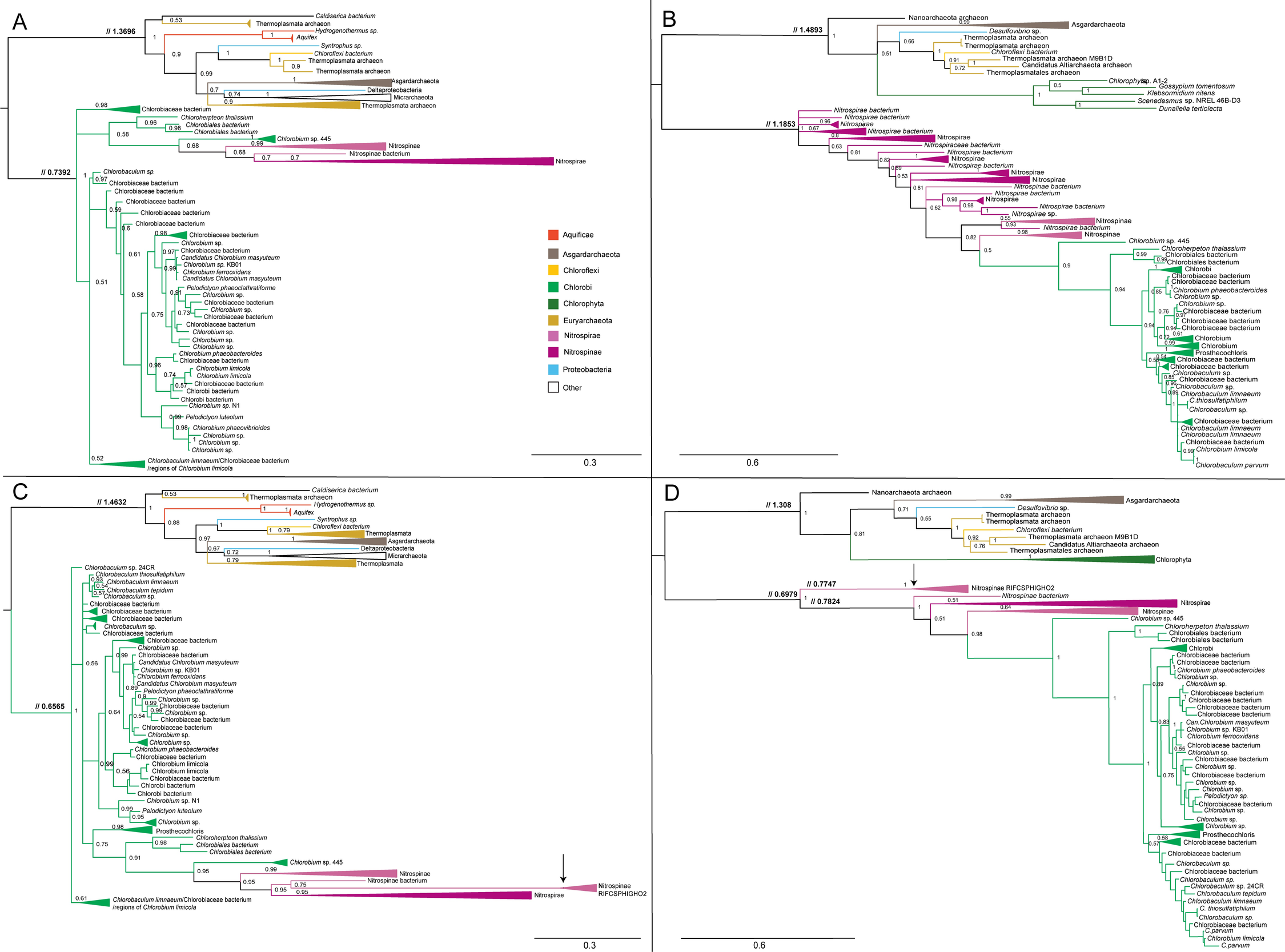

Supplement: S3 Fig — Trees depicted without (A, B) and with (C, D) Nitrospinae metagenomic sequences from groundwater metagenomic biosampling included in the alignment depicted with arrow. Collapsed clades labeled with taxonomic group names. Support values show consensus posterior probabilities. (TIF) [file pone.0275539.s004.tif]
