# Supplementary material for: Chimeric inheritance and crown-group acquisitions of carbon fixation genes within Chlorobiales: Origins of autotrophy in Chlorobiales and implication for geological biomarkers
Source: PLoS One. 2022 Oct 13;17(10):e0275539. doi: 10.1371/journal.pone.0275539 (PMC9560492; doi:10.1371/journal.pone.0275539)
Supplement: Attachment — Submitted filename: Paoletti & Fournier Report.pdf [file pone.0275539.s005.pdf]

The manuscript of Paoletti and Fournier explores the phylogenetic history of the reverse TCA cycle in the order *Chlorobiales*. Biomarkers, mostly derived from aromatic carotenoids once believed to be specific markers for phototrophiic sulfur bacteria, have been oberved in the rock record extending back about 1.64 Ga. However, recent studies have shown that aromatic carotenoids are also produced by many modern-day cyanobacteria. To properly interpret isotopic fractionation of carbon in biomarkers requires knowledge of the carbon fixation pathways in use by organisms that produced the biomarkers. Thus, this is a timely study that will be of interest to a wide audience. Comments for the authors follow.

1. Lines 31 to 33. Considering that renieratene and isorenieratene have recently been shown to be produced by cyanobacteria that can also produce synechoxanthin, okenone may be the only reliable biomarker associated with (purple) sulfur bacteria. Although chlorobactene has not been shown to be produced by cyanobacteria yet, any cyanobacterium that can synthesize isorenieratene could in principle make chlorobactene, assuming the carotene desaturase could function with gamma-carotene as substrate.
2. Lines 50-51. The CBB cycle also occurs in many chemolithoautotrophic bacteria, not just photoautotrophs.
3. Lines 71-73. I am not sure of the "official" state of taxonomy concerning the order *Chlorobiaceae*, but I think there are only three genera (see Imhoff 2003) now: *Prosthecochloris*, *Chlorobium* and *Chlorobaculum*. *Pelodictyon* (note spelling) and *Ancalochloris* are no longer considered to be valid taxa. The status of *Chloroherpeton* is less clear, but it is probably the type genus of a separate family, because *Chloroherpeton* are metabolically distinct from all other green sulfur bacteria in that they can only oxidize sulfide to the level of sulfur/polysulfide.
4. Lines 73-75. The authors should probably cite Liu et al., 2012, *Front. Microbiol.* **3**, 185. *Ignavibacterium album* is an aerobe that can grow as a facultative or fermentative anaerobe as well. It has a full aerobic electron transfer chain and has been demonstrated to grow by aerobic respiration.
5. Lines 77 to 79. *Thermochlorobacter* spp. has been cultured but not axenically, only in enrichment cultures (Tank et al. 2017). It is clearly a full aerobe with a complete electron transfer chain to match, with metabolic properties similar to those of *Chloroacidobacterium*. *Thermochlorobacter* and related taxa are unable to oxidize sulfide at all, do not fix carbon dioxide, but have a photosynthetic apparatus similar to that of GSB. These bacteria are not "sulfur bacteria" at all, unless assimilatory sulfate reduction is included in the definition, but then just about all bacteria would be sulfur bacteria.
6. Lines 71 to 81. The authors do not present any analysis of the current state of the 16S rRNA phylogeny of GSB, nor do they present any phylogenetic trees based upon shared proteins among these organisms. This makes the interpretation of the trees presented for the rTCA cycle enzymes difficult for those less familiar with the taxonomy of green bacteria. A 16S rRNA tree finally appears in Figure 11.
7. The importance of the last several points is that the earliest diverging members of the *Chlorobiales* and the sister taxon *Ignavibacteria* is that these organisms were aerobes, not anaerobes as once generally believed. This has important implications for how the authors interpret their trees and the information derived from them. Even if the authors do not agree with these data, they must present the context for their intepretation, so I would suggest

that the authors begin with a section describing protein and 16S rRNA trees to establish the relationships of the organisms under consideration with other bacteria.

8. Lines 109-111. Again, considering that I. album and Thermochlorobacter are aerobes, and that all GSB have retained the genes for at least one terminal cytochrome/quinol oxidase, it seems likely that the GSB are derived from aerobic ancestors that gained phototrophy and many other traits based on HGT.
9. Figure 3. *Chloracidobacterium thermophilum* is not an autotroph. It is a microaerophilic heterotroph that mostly consumes amino acids, especially branched chain amino acids. It likely uses the partial reverse TCA cycle to carboxylate succinyl-CoA to make 2-oxoglutarate.
10. Line 181. Not exactly. *Thermochlorobacter* doesn't have fumarate reductase because it has an oxidative TCA cycle, not a reductive one.
11. Figure 5B. This figure largely replicates what I expect a 16S rRNA tree would look like, and probably a tree based on concatenated core proteins as well. In panel A, I am not sure whether there are any autotrophic *Acidobacteria*, but I don't think there are any.
12. Lines 206-210. Organisms that can degrade branched chain amino acids as carbon and nitrogen sources can do so by making succinate/succinyl-CoA. This can be carboxylated to produce 2-oxoglutarate by the partial reverse TCA cycle to supply precursors for Chl/BChl biosynthesis. This is very beneficial to organisms that are unable to grow as full autotrophs.
13. Figure 6A. Not all *Chlorobiales* are autotrophs (see panel B below).
14. Line 218 to 225. Presumably this refers to Figure 6, but panel 6B looks like a 16S rRNA tree to me with early diverging *Chloroherpeton* and *Thermochlorobacter* sequences. What is misrooted about this?
15. Figure 9A. *Chlorobium* sp. 445 should be orange, not green (see panel B).
16. Lines 305 to 307. Seems that the ancestral state was an oxidative rather than a reductive TCA cycle, so gain more likely than loss?
17. Lines 330 to 332. Is pyruvate dehydrogenase present in the aerobic heterotrophs?
18. Figure 11. This is an interesting figure, but it could perhaps be made still more informative by including some other events as well. For example, all the organisms in the gray shading acquired the genes for type-1 reaction centers, FMO bacteriochlorophyll *a* binding protein, and the genes for bacteriochlorophyll *c* biosynthesis and chlorosome assembly. The *Chlorobiaceae* gained the ability to oxidize sulfur/polysulfide to sulfate, and *Chlorobaculum* gained the ability to oxidize thiosulfate to sulfate.
19. Most (but not all) members of the *Chlorobiaceae* can synthesize chlorobactene or isorenieratene; a few seem to have lost CrtQ. *Chloroherpeton* and *Thermochlorobacter* cannot make aromatic carotenoids, and that seems to be trait that was acquired relatively late by GSB. However, exactly when this trait was acquired is not clear. All strains in the shaded area, as well as *I. album*, can synthesisize carotenoids, which are essential for organisms that use chlorophylls to perform phototrophy in the presence of oxygen.
