# Supplementary material for: Chimeric inheritance and crown-group acquisitions of carbon fixation genes within Chlorobiales: Origins of autotrophy in Chlorobiales and implication for geological biomarkers
Source: PLoS One. 2022 Oct 13;17(10):e0275539. doi: 10.1371/journal.pone.0275539 (PMC9560492; doi:10.1371/journal.pone.0275539)

Sept 10, 2022

To the Editor,

Response to Reviewer #1 Comment: The authors have done an excellent job of addressing my extensive comments, with only a single exception. I don't think Chloroherpeton belongs in the Chlorobiaceae. It should be a 2nd family, Chloroherpetonaceae, in the Chlorobiales. Other than this, I am satisfied with the modifications made to the original manuscript, which I believe has been improved.

We thank reviewer one for this point. After considering newer literature on the classification system for Chlorobi along with NCBI taxonomic identifications, we have decided to place Chloroherpeton in the family Chloroherpetonaceae, separate from Chlorobiaceae. Besides altering taxonomic names in the manuscript where required and relabeling Figure 12, we believe this change does not further alter our findings or conclusions.

Sincerely,

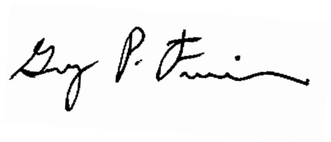

Gregory P. Fournier (corresponding author)

g4nier@mit.edu

Madeline Paoletti, mpaolett@mit.edu

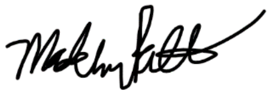

Supplement: Attachment — Submitted filename: Response to Reviewers.pdf [file pone.0275539.s007.pdf]
